# Human milk oligosaccharides and the association with microbiota in colostrum: a pilot study

**DOI:** 10.1007/s00203-023-03787-3

**Published:** 2024-01-08

**Authors:** Wen Sun, Lin Tao, Chen Qian, Peipei Xue, Xiankun Tong, Li Yang, Fang Lu, Hua Wan, Yingna Tao

**Affiliations:** 1 Department of Traditional Chinese Medicine, Shanghai Fourth People’s Hospital Affiliated to Tongji University, Shanghai, 200434 China; 2 Laboratory of Immunopharmacology, Shanghai Institute of Materia Medical, Chinese Academy of Sciences, Shanghai, 201210 China; 3 Department of Gynaecology and Obstetrics, Shanghai Fourth People’s Hospital Affiliated to Tongji University, Shanghai, 200434 China; 4 Department of Breast, Shuguang Hospital Affiliated to Shanghai University of Traditional Chinese Medicine, Shanghai, 201203 China

**Keywords:** Human milk oligosaccharides, Maternal characteristics, Microbiota

## Abstract

HMOs (Human milk oligosaccharide) has an impact on maternal and infant health. Colostrum samples of 70 breastfeeding women in China were collected and recorded clinical characteristics. The major oligosaccharides and microbiota were quantitated in colostrum. The concentration of fucosylated HMOs in primipara was higher than that of multipara (*p* = 0.030). The concentration of N-acetylated HMOs in vaginal delivery milk was less than that of cesarean (*p* = 0.038). Non-fucosylated HMOs of breastfeeding women were less than that of breast pump (*p* = 0.038). Meanwhile, the concentration of LNT was positively correlated with *Lactobacillus* (*r* = 0.250, *p* = 0.037). DS-LNT was negatively correlated with *Staphylococcus* (*r* = – 0.240, *p* = 0.045). There was a positive correlation of *Streptococcus* with LNFP II (*r* = 0.314, *p* = 0.011) and 3-SL (*r* = 0.322, *p* = 0.009). In addition, there was a negative correlation between 2'-FL and 3-FL (*r* = – 0.465, *p* = 0.001). There was a positive correlation between LNT and LNnT (*r* = 0.778, *p* = 0.001). Therefore, the concentration of HMOs is related to number of deliveries, delivery mode, lactation mode and perinatal antibiotic. The concentration of HMOs is related to *Lactobacillus*, *Streptococcus* and *Streptococcus* in colostrum. In addition, there are connections between different oligosaccharides in content. The study protocol was also registered in the ClinicalTrails.gov (ChiCTR2200064454) (Oct. 2022).

## Introduction

Human milk, which is the biological norm of infant nutrition, has been reported to contain many oligosaccharides. The content of HMOs (Human milk oligosaccharide) in breast milk is second to lactose and lipids (Thum et al. 2021). Colostrum is usually a sticky yellowish liquid secreted by the mammary gland within 7 days after delivery. The content of HMOs in colostrum is more than that of mature milk (Donovan and Comstock 2016). The concentration of HMOs in colostrum is about 9–22 g/L, decreased with the duration of lactation (Samuel et al. 2019; Liu et al. 2021; Poulsen et al. 2022). As we all know, HMOs has important physiological functions. It can affect the health status of newborns by supplementing probiotics (Pamela et al. 2018; Triantis et al. 2018; Sakanaka et al. 2019), resisting harmful microbiota (Quinn et al. 2020), simulating intestinal epithelial cell binding ligands and regulating the immune response (Ayechu-Muruzabal et al. 2022). Infant formula containing 2'-FL has been approved by the European Union and the US Food and Drug Administration (Reverri et al. 2018).

So far, 200 kinds of HMOs have been found (Urashima et al. 2018). At least 157 different HMOs structures were isolated and identified (Oursel et al. 2017; Peterson and Nagy 2021). The diversity of HMOs is affected by glycosidic bonds of different sugar units. According to the substituents, oligosaccharides are mainly divided into fucosylated HMOs, sialylated HMOs and non-fucosylated HMOs (Samuel et al. 2019). The main fucosylated HMOs are 2'-fucosyllactose (2'-FL) and 3-fucosyllactose (3-FL). Sialylated HMOs mainly includes 3-sialyllactose (3'SL) and 6-sialyllactose (6'SL). Non-fucosylated HMOs mainly include lacto-N-tetraose (LNT) and lacto-N-neotetraose (LNnT).

The concentration and composition of HMOs vary from individual to individual and from lactation to lactation (Thum et al. 2021). Fucosyltransferase 2 and 3 are encoded by secretory and Lewis genes, respectively. There are genetic variations that affect the activity (Soyyılmaz et al. 2021). In addition to genetic factors, other maternal factors may affect the composition of breast milk to some extent. The composition of HMOs was examined in 290 healthy breast milk samples. There were differences in the concentration of HMOs before pregnancy, delivery and parity (Samuel et al. 2019). Studies have provided data to show the correlation between HMOs and maternal factors, such as body weight, BMI, parity and age (McGuire et al. 2017; Azad et al. 2018). On the other hand, HMOs may also affect lactating mothers. HMOs can be used as a probiotic or antimicrobial agent to affect Staphylococci, Streptococci, *Lactobacillus* and Enterococci (Bode 2012). These oligosaccharides may affect the bacterial community in milk by promoting the growth of specific genera (Hunt et al. 2012), or directly regulate breast epithelial cell response and local immune response (Bode 2012).

Human milk microbiota affects maternal and infant health through breastfeeding. At the same time, HMOs affect the distribution and growth of infant intestinal microbiota. HMOs exist in human milk, which are associate with the milk microbiota and mother’s situation. Understanding the biological and environmental factors associated with the HMOs is an important part of the complex subject of maternal and child health. However, there are few studies on the relationship of HMOs and milk microbiota in China, especially in colostrum.

To our knowledge, the relationship between clinical characteristics and high levels of nutrition (including HMOs) in breast milk has not been confirmed. Though HMOs has been proved to have an impact on infant intestinal microbiota, it is not clear whether HMOs affected the microbiota of milk. Therefore, we collected human colostrum and pioneered the exploration of the potential relationship between HMOs and microbiota. At the same time, we observed the correlation of HMOs and maternal clinical characteristics. The findings may enrich the clinical data of breast milk research and guidelines for maternal and child health.

## Materials and methods

### Study participants

Healthy Chinese lactating women from the Shanghai Fourth People’s Hospital Affiliated to Tongji University School of Medicine were included in the study from October 2022 to December 2022. Informed consent was obtained from all subjects at enrollment. The study was approved by the Institutional Review Board (IRB) of the Shanghai Fourth People’s Hospital (20,211,124-001).

The inclusion criteria for the participants were as follows: (I) they were healthy and lactating; (II) postpartum colostrum was collected within 5 days after delivery. Exclusion criteria were as follows: (I) there was mammary abscess, (II) there was any other mammary pathology.

### Collection and processing of milk samples

Colostrum samples were collected from healthy breastfeeding women within 5 days after delivery and 5 ml of milk was collected from each side of the breast. Women were asked to have not fed or expressed from the study breast for at least 2 h prior to sample collection. Before the milk collection, the areola and nipple area were sterilized by using 75% alcohol. The first drop of milk (about 150 μl) was discarded to avoid contamination. Samples were collected using a single-use, sterile containers and stored at − 20 °C. Microbiotal DNA was extracted within 24 h after collection.

### Human milk oligosaccharide analysis

The HMO analysis was performed as previously described in the Laboratory of Immunopharmacology, Shanghai Institute of Materia Medical. After the samples were defatted and protein removed, the oligosaccharides were reduced to aldol with 1.0M sodium borohydride (NaBH4) and cleaned by solid phase extraction (SPE) with graphitized carbon cartridges. LC separation using binary gradient at 0.2 mL/min flow rate for 55 min. MS analysis runs in forward mode. Raffinose was added to each milk sample as an internal standard for absolute quantification. The concentration of HMOs was calculated as the specific oligosaccharides detected. The following HMOs were detected based on retention time comparison with commercial standard oligosaccharides (Zhenzhun Biotechnology, Shanghai, China) and mass spectrometry analysis: 2ʹ-fucosyllactose (2ʹ-FL Cat.ZMI-108106), 3-fucosyllactose (3-FL Cat.ZG10022), 3ʹ-sialyllactose (3ʹ-SL Cat.ZG-10015), lacto-N-tetraose (LNT Cat.ZEO-GLY-010), difucosyl-LNT (DS-LNT Cat. ZEO-GLY-066), lacto-N-neotetraose (LNnT Cat.ZB-038728), lacto-N-fucopentaose I (LNFP I Cat. ZG10048), LNFP II (Cat. ZG10049), and LNFP III (Cat. ZG10051).

### Microbiotal DNA extraction

Total microbiotal DNA was extracted from the colostrum and mastitis milk samples using a microbiotal DNA Extraction Kit (TIANGEN, Beijing, China). Initially, milk samples (1 mL) were centrifuged at 12,000 rpm for 20 min at 4 °C. The supernatants with the fat and whey layer were removed. The protocol included an initial rupture of the microbiotal wall by 30 min of incubation with lysozyme (20 mg/mL) at 37 °C. Total DNA was then isolated from the pellets using the microbiotal DNA extraction kit (TIANGEN Cat: DP302) following the manufacturer’s instructions. A spectrophotometer (Thermo NanoDrop) was used to quantify the DNA. The purified DNA extracts were stored at – 20 °C.

### Microbiotal quantitation of colostrum

Standard curves were created using serial tenfold dilutions of microbiotal DNA extracted from qPCR amplification products (Collado et al. 2009). A strain belonging to each of the microbiotal genera or groups targeted in this study was used to construct the standard curve. PCR standards were added, and the standard curve was obtained.

Microbial quantitation was based on the conserved marker gene 16s rRNA for microbiotal genus including *Bifidobacterium*, *Lactobacillus*, *Staphylococcus* and *Streptococcus*. Real-time PCR quantitation for target microbiotal gene was conducted with ABI 7900HT Fast Real-time PCR System (Applied Biosystems, Thermo Fisher, U.S.). Each reaction mixture (25 μl) was composed of 12.5 μl SYBR Green Master Mix (TIANGEN Cat: FP205), 0.1 ul of each of the specific primers at a con centration of 100 μM and 5.0 μL of template (1 ng/μl). Forty cycles of two-step polymerase chain reaction amplification were performed on the Applied Biosystems real-time polymerase chain reaction system (95 °C 5 s, 60 °C 32 s). The microbiotal concentration in each sample was measured as log_10_ genome equivalents by the interpolation of the Ct values obtained by the milk samples into the standard calibration curves. All samples were analyzed in two independent PCR assays, and the standard curve should be determined at all times.

The primer sequence was as follows: (*Lactobacillus*) LactoF TGGAAACAGRTGCTAATACCG; LactoR GTCCATTGTGGAAGATTCCC; (*Bifidobacterium*) T-Bifid426-F CTCGTAGGCGGTTCGTC; T-Bifid426-R GAACATGTCAAGCCCAGG; (*Staphylococcus*) TStaG422 GGCCGTGTTGAACGTGGTCAAATCA; TStag765 TACCATTTCAGTACCTTCTGGTAA;(*Streptococcus*) Tuf-Strep-1 GAAGAATTGCTTGAATTGGTTGAA; Tuf-Strep-R GGACGGTAGTTGTTGAAGAATGG.

### Statistical analysis

All data were expressed as $\overline{x }\pm s$ or [M (Q25, Q75)]. Continuous variables were compared using the *t* test or the Mann–Whitney *U* test. A single-factor analysis of variance (ANOVA) or Kruskal–Wallis test was used for multiple comparisons. A spearman correlation matrix was calculated for the HMO groups and the different microbiota, as well as individual HMO structures and the microbiota. *p* < 0.05 was considered the statistically significant level. All the PCR data represented three groups. The statistical analysis was done with IBM SPSS version 27.0 (Chicago, IL, U.S.) and Graphpad Prism version 9.0 (La Jolla, CA, U.S.).

## Results

### Clinical data of participants

**Fig. 1:**
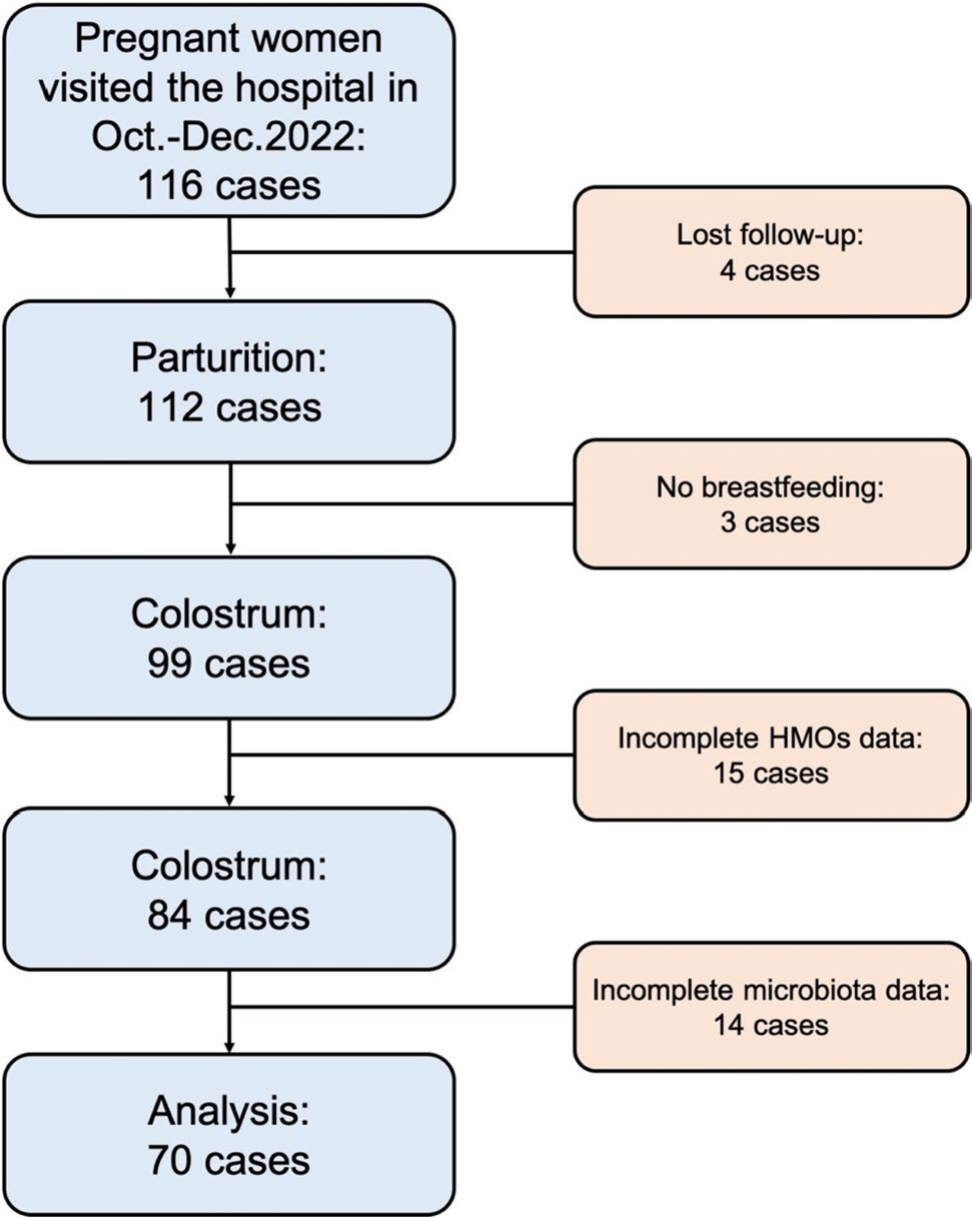
Clinical trial flow chart

A total of 70 subjects were included in this study, excluding those who lost follow-up and missing data (Fig. 1). Table 1 shows the participant’s description data. The average age of pregnant women was 29.7 ± 3.9, and the gestational weeks were normal. The maternal colostrum with an average of 2.9 ± 1.0 days was collected. The subjects were counted according to the number of deliveries, mode of delivery, lactation mode, situation before delivery, and whether to use antibiotics in perinatal period.

**Table 1:** Clinical data of 70 healthy participants [$\overline{x }\pm s$ or *n* (%)]

	$\overline{\chi }\pm s$ or *n* (%)
Age	29.7 ± 3.9
Days after delivery	2.9 ± 1.0
Weeks of pregnancy	39.0 ± 1.32
Mode of delivery	
Vaginal	33 (47.1)
Cesarean	37 (52.9)
Number of deliveries	
Primipara	45 (64.3)
Multipara	25 (35.7)
Whether to use antibiotics in the perinatal period	
Antibiotics	39 (55.7)
Without antibiotics	31 (44.3)
Lactation mode	
Breastfeeding	59 (84.3)
Breast pump	11 (15.7)
Situation before delivery	
Work	15 (21.4)
Vacation	55 (78.6)

### The concentration of HMOs

**Fig. 2:**
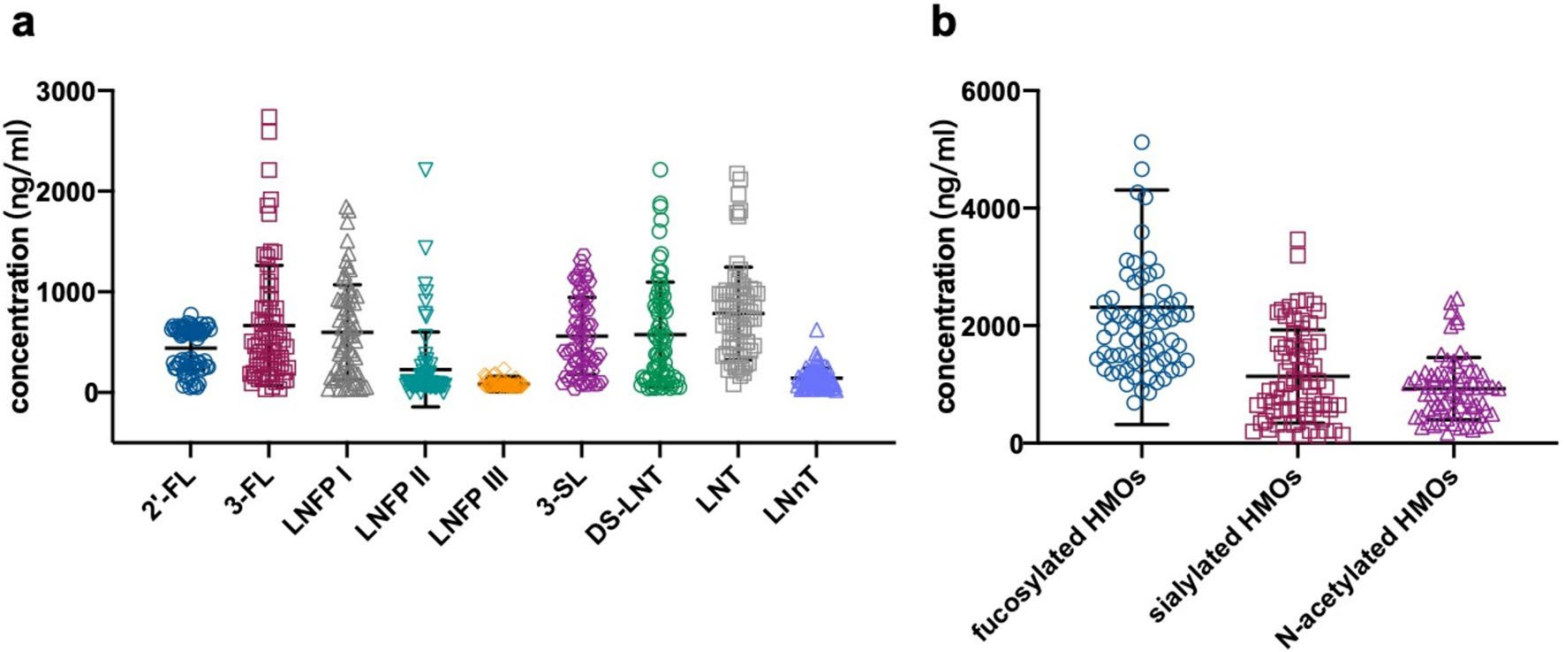
The concentrations of HMOs in human colostrum (**a** the concentrations of several HMOs: 2'-FL 440.1 ± 215.6 ng/ml, 3-FL 665.7 ± 597.8 ng/ml, LNFP I 597.3 ± 470.1 ng/ml, LNFP II 373.4 ± 228.5 ng/ml, LNFP III 84.3 ± 33.9 ng/ml, 3-SL 560.7 ± 383.9 ng/ml, DS-LNT 575.6 ± 522.7 ng/ml, LNT 785.7 ± 460.5 ng/ml, LNnT 142.7 ± 99.4 ng/ml; **b** the concentrations of three kinds of HMOs: fucosylated HMOs 2314.3 ± 1994.4 ng/ml, sialylated HMOs 1136.3 ± 791.5 ng/ml, non-fucosylated HMOs928.3 ± 530.7 ng/ml)

**Fig. 3:**
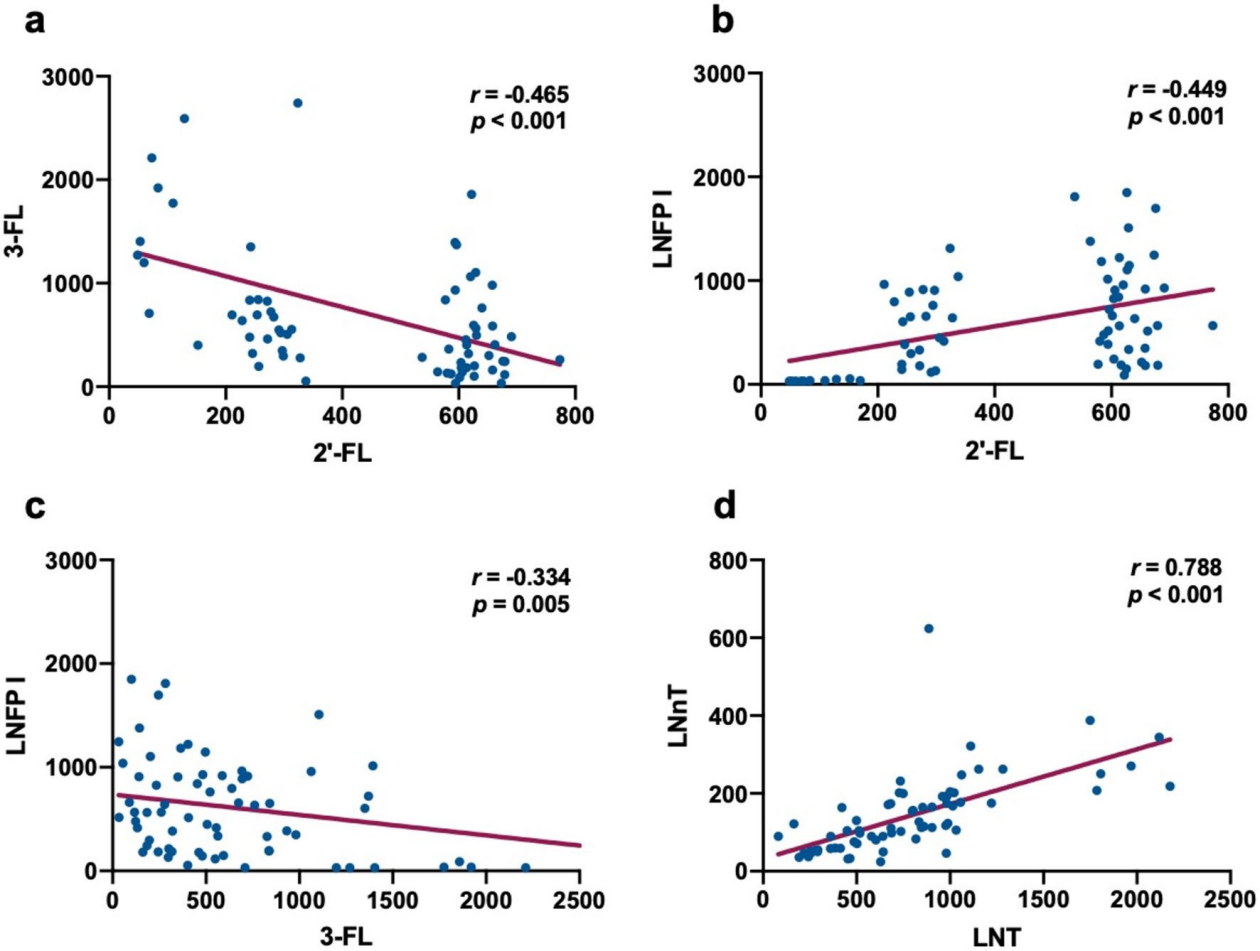
The correlation between HMOs in milk (**a** 2'-FL and 3-FL; **b** 2'-FL and LNFP I; **c** 3-FL and LNFP I; **d** LNT and LNnT)

The concentrations of major oligosaccharides were as follow: 2'-FL 440.1 ± 215.6 ng/ml, 3-FL 665.7 ± 597.8 ng/ml, LNFP I 597.3 ± 470.1 ng/ml, LNFP II 373.4 ± 228.5 ng/ml, LNFP III 84.3 ± 33.9 ng/ml, 3-SL 560.7 ± 383.9 ng/ml, DS-LNT 575.6 ± 522.7 ng/ml, LNT 785.7 ± 460.5 ng/ml, LNnT 142.7 ± 99.4 ng/ml (Fig. 2). The substituents are divided into three categories according to their substituents, which are fucosylated HMOs (2ʹ-FL, 3ʹ-FL, LNFP I, LNFP II, LNFP III), sialylated HMOs (3-SL, DS-LNT) and non-fucosylated HMOs (LNT, LNnT). Furthermore, the correlation between different oligosaccharides was analyzed (Table 2). The results showed that there was a negative correlation between 2'-FL and 3-FL (*r* = – 0.465, *p* = 0.001), 3-FL and LNFP I (*r* = – 0.334, *p* = 0.005). There was a positive correlation between 2'-FL and LNFP I (*r* = 0.449, *p* = 0.001), LNT and LNnT (*r* = 0.778, *p* = 0.001) (Fig. 3).

**Table 2:** The correlation between HMOs in milk (*p < 0.05)

	2'-FL	3-FL	LNFP I	LNFP II	LNFP III	DS-LNT	3'-SL	LNT	LNnT
2'-FL									
* r*	1								
* p*	–								
3-FL									
* r*	– 0.465*	1							
* p*	0.001	–							
LNFP I									
* r*	0.449*	– 0.334*	1						
* p*	0.001	0.005	–						
LNFP II									
* r*	– 0.418*	0.669*	– 0.010	1					
* p*	0.001	0.001	0.938	–					
LNFP III									
* r*	– 0.151	0.430*	0.392*	0.553*	1				
* p*	0.212	0.001	0.001	0.001	–				
DS-LNT									
* r*	0.189	0.093	0.569*	0.066	0.512*	1			
* p*	0.117	0.445	0.001	0.585	0.001	–			
3'-SL									
* r*	– 0.14	0.215	0.351*	0.351*	0.499*	0.509*	1		
* p*	0.246	0.074	0.003	0.003	0.001	0.001	–		
LNT									
* r*	– 0.190	0.271*	0.211	0.541*	0.419*	– 0.259*	0.087	1	
* p*	0.116	0.023	0.079	0.001	0.001	0.030	0.475	–	
LNnT									
* r*	– 0.232	0.277*	0.392*	0.562*	0.643*	0.073	0.403*	0.788*	1
* p*	0.054	0.020	0.001	0.001	0.001	0.548	0.001	0.001	–

### The Relationship between HMOs and clinical characteristics

**Fig. 4:**
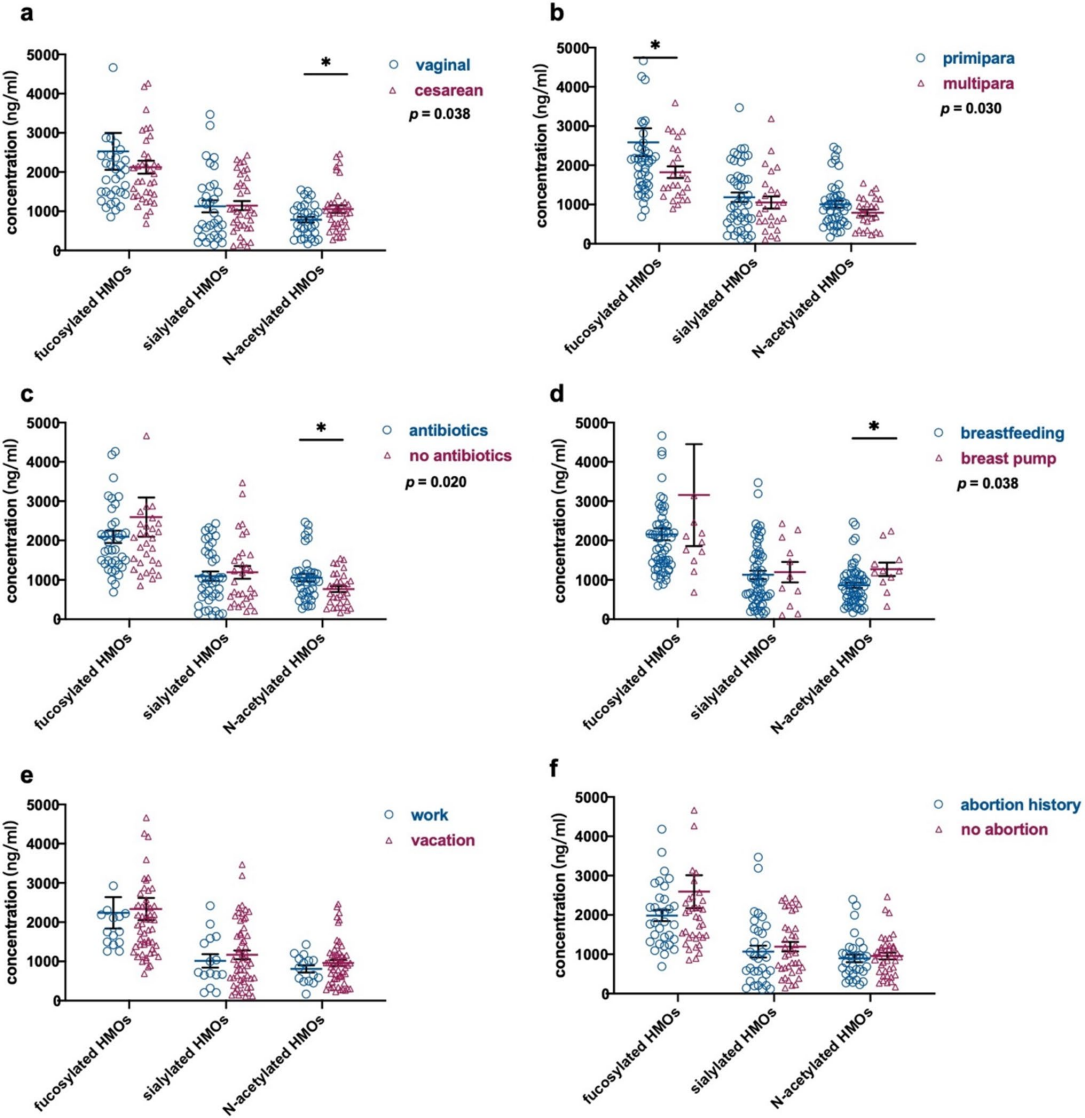
Clinical characteristics and HMOs of subjects (**a** mode of delivery; **b** number of deliveries; **c** antibiotics in perinatal period; **d** lactation mode; **e** situation before delivery; **f** abortion history; *p < 0.05)

The HMO of different groups are compared according to the number of deliveries, mode of delivery, lactation mode, situation before delivery, and whether to use antibiotics in perinatal period (Fig. 4). The results showed that the concentration of non-fucosylated HMOs in vaginal delivery milk was less than that of cesarean (*p* = 0.038). The concentration of primipara Fucosylated HMOs was higher than that of multipara (*p* = 0.030). Non-fucosylated HMOs in breast milk of women who took antibiotics during the perinatal period were less than those who did not use (*p* = 0.020). Non-fucosylated HMOs in breast milk from breastfeeding women who were sucked by the baby were less than that of that breast pump (*p* = 0.038). Vacation or work before delivery and abortion history has no significant effect on HMOs concentration (*p* > 0.05).

### The correlation between HMOs and microbiota

**Fig. 5:**
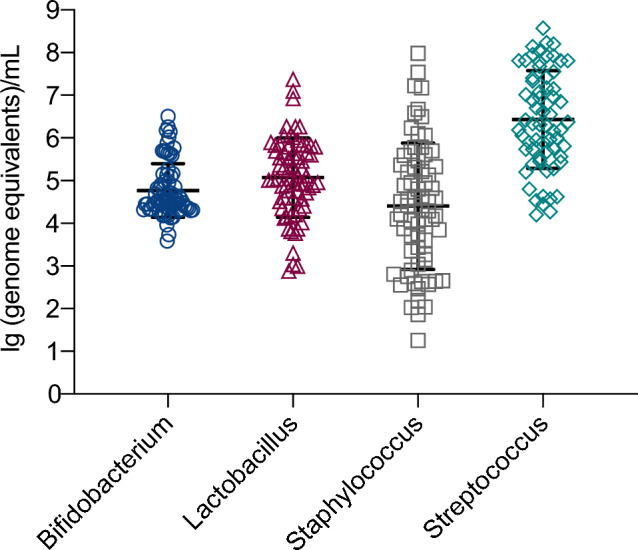
Concentration of microbiota in human colostrum (*Bifidobacterium* 4.77 × 10^^4^ ± 0.63 × 10^^4^/ml, *Lactobacillus* 5.07 × 10^^4^ ± 0.93 × 10^^4^/ml, *Staphylococcus* 4.40 × 10^^4^ ± 1.48 × 10^^4^/ml, *Streptococcus* 6.43 × 10^^4^ ± 1.45 × 10^^4^/ml)

**Fig. 6:**
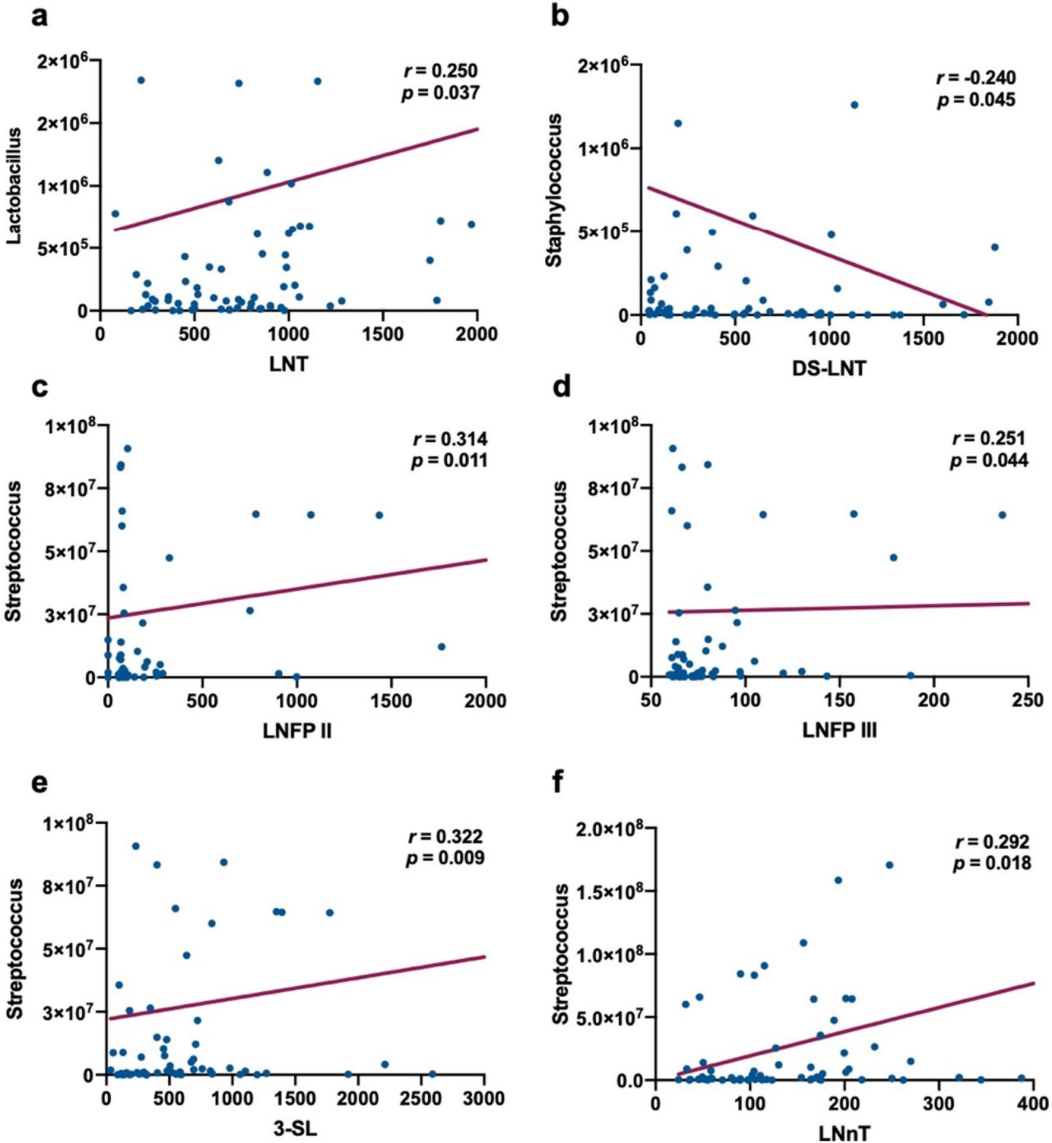
The correlation between microbiota and HMOs (**a** LNT and *Lactobacillus*; **b** DS-LNT and *Staphylococcus*; **c** LNFP II and *Streptococcus*; **d** LNFP III and *Streptococcus*; **e** 3-SL and *Streptococcus*; **f** LNnT and *Streptococcus*)

*Bifidobacterium*, *Lactobacillus*, *Staphylococcus* and *Streptococcus* was detected in milk (Fig. 5). We analyzed the correlation between microbiota and HMOs (Fig. 6, Table 3). The results showed that LNT was positively correlated with *Lactobacillus* (*r* = 0.250, *p* = 0.037). The concentration of DS-LNT was negatively correlated with *Staphylococcus* (*r* = – 0.240, *p* = 0.045). There was a positive correlation between *Streptococcus* and LNFP II (*r* = 0.314, *p* = 0.011), LNFP III (*r* = 0.251, *p* = 0.044), 3-SL (*r* = 0.322, *p* = 0.009), LNnT (*r* = 0.292, *p* = 0.018).

**Table 3:** The correlation between microbiota and HMOs

	2'-FL	3'-FL	LNFP I	LNFP II	LNFP III	DS-LNT	3-SL	LNT	LNnT
*Bifidobacterium*									
* r*	– 0.215	0.069	0.080	0.073	0.170	0.167	0.223	0.081	0.175
* p*	0.074	0.569	0.512	0.551	0.159	0.168	0.064	0.507	0.147
*Lactobacillus*									
* r*	– 0.019	0.139	– 0.098	0.068	0.074	– 0.201	– 0.091	0.250*	0.116
* p*	0.874	0.252	0.420	0.575	0.544	0.095	0.454	0.037	0.340
*Staphylococcus*									
* r*	0.029	– 0.021	– 0.151	– 0.056	– 0.068	– 0.240*	– 0.085	0.206	0.023
* p*	0.809	0.860	0.213	0.647	0.576	0.045	0.485	0.087	0.848
*Streptococcus*									
* r*	– 0.102	0.200	0.192	0.314*	0.251*	0.219	0.322*	0.162	0.292*
* p*	0.421	0.110	0.126	0.011	0.044	0.080	0.009	0.197	0.018

*p < 0.05

## Discussion

In this study, we collected the precious colostrum. We quantitatively analyzed oligosaccharides in colostrum. Then, we found the relevance of oligosaccharides and the clinical characteristics of breastfeeding women. Furthermore, this study explored the relevance of the microbiota and HMOs in milk, which could guide lactation.

Human milk contains the plenty HMOs and various microbiota categories. The results from our study confirmed that colostrum samples of human milk consisted of high content of HMOs, with the evidences indicating that the Fucosylated HMOs as the highest compared with Sialylated HMOs or non-fucosylated HMOs. It was found that the ratio of the oligosaccharides in HMOs varied among the breastfeeding (Thurl et al. 2017; Xu et al. 2017). Our study exhibited the concentrations of 2'-FL, 3-FL, LNT and LNFP I were 0.58 g/L, 0.4 g/L, 0.55 g/L and 0.47 g/L correspondingly. It was reported that 2'-FL could be considered as the most abundant oligosaccharide in colostrum with the level almost 4.1 g/L, with the concentrations of 3-FL, LNT and LNFP I lower than it (Marriage et al. 2015; Thum et al. 2021). Meanwhile, the results from one meta study indicated that the mean content of 2'-FL, 3-FL and LNFP I varied from 0.14 to 2.74 g/L (Thurl et al. 2017), which was consistent with our founding. The concentrations of LNFP II and LNFP III were 0.19 g/L and 0.07 g/L, which were lower than the reported mean content of 0.21–0.58 g/L (Thurl et al. 2017). Previously research indicated that the level of LNnT was 0.36–1.12 g/L (Ma et al. 2018) which was higher than our results of 0.12 g/L. And the concentrations of 3-SL and DS-LNT were 0.50 g/L and 0.39 g/L in this study, which were different from the reported results of 0.19–0.29 g/L (3-SL) and 0.50–0.77 g/L(DS-LNT) (Gidrewicz and Fenton 2014; Van Niekerk et al. 2014). Previously studies mainly concentrated in the European or America, which rarely involved Asia, especially the China. Differences between our results and other reports would originate in the regional differences, which would be affected by the race, culture and living habits, etc. Besides, the concentrations of HMOs would be influenced by the personal status, including the α (1,2)-fucosyltransferase (FUT2) and enzyme FUT3 encoded by Lewis blood group gene (Morrow et al. 2011; Soyyılmaz et al. 2021). In addition, the differences would be attributed to the measurements conducted in the various studies (Austin and Bénet 2018; Huang et al. 2019). The method conducted in our results was LC–ESI–MS to determine the concentrations with the advancement of fast and convenient (Chaturvedi et al. 1997; Porfirio et al. 2020; Catenza and Donkor 2021). Other detection methods, such as refractive index detection (RID), evaporative light scattering detection (ELSD) and capillary electrophoresis (CE), have common disadvantages including complicated sample preparation steps and low sensitivity (Sarkozy et al. 2021).

The relationship between kinds of HMOs was observed in our study, including the negative correlation between 2'-FL and 3-FL which was consistent with the reported results (*r* = 0.78–0.99) (Austin et al. 2016; Thurl et al. 2017). The two HMOs contained the similar molecular structure which shared the same substrate (Guanosine 5′-diphosphate (GDP)-l-amylose), further resulting the negative correlation (Thum et al. 2021). The positive relationship of 2'-FL and LNFP I was observed in this study, which originated in α1-2 Rockweed glycosylation and the high dependence of FUT2 activity (Phipps et al. 2021). The results from our study also indicated that LNT positively related with LNnT, with supporting evidences that the two HMOs could be regulated by α1-2-fucosyltransferase (FUT2) (Sprenger et al. 2017). In addition, it was reported that LNT was negatively related with 2′-FL (Sprenger et al. 2017), which was different from our results that no relationship between LNT and 2′-FL founded. Meanwhile, our results exhibited that LNFP I was negatively related with 3-FL. However, Gabrielli et al. (2011) reported a positive correlation between the two HMOs, which would attributed to the different race and living habits, etc. Based on the above results, it was supposed that the HMOs with the similar molecular structure contained the homologous synthesis pathway, such as the regulation of Sialylated HMOs including 3'-SL and DS-LNT.

Oligosaccharides were formed through the conjunction of nucleotide sugar molecules based on the glycosidic bonds, which were catalyzed by the glycosyltransferases. The generation of HMOs was regulated by the cellular glycosyltransferases (including FUT2 and FUT3) in mammary gland cells, and simultaneous influenced by substrate. All the process were closely related with the physiological state of the mother, which caused the sightly influence on the glycosylation during the onset of the breastfeeding (Samuel et al. 2019). Concentrations of specific HMOs were related with maternal age, allergy history, pre-pregnancy body mass index, gestational age, mode of delivery, infant gestational age and sex (Wang et al. 2020). The results from this study indicated that higher concentrations of fucosylated HMOs in first-time mothers’ milk was founded compared with the milk from multiple births. Niekerk et al. (2014) also reported that the concentrations of HMOs were negatively correlated with births and the first contain the higher level, which was consistent with our results. But Azad et al. (2018) reported that there were relationships between the concentrations of HMOs and the times of birth. Meanwhile, this study exhibited that the non-fucosylated HMOs concentrations in breast milk from the mother underwent vaginal delivery were lower than it from the mother with cesarean delivery. However, it was reported that no relationship was founded between HMOs concentrations and delivery modes (Azad et al. 2018). In addition, the non-fucosylated HMOs concentrations in breast milk from breastfeeding mothers who are suckled by their babies was lower than it from the mothers who breastfeed the children through the breast-pumps. And the concentrations of non-fucosylated HMOs in the mother who unused antibiotics during the perinatal administration were higher. All the evidences suggested that there are differences in the susceptibility of various HMOs to maternal characteristics, further revealed that there may be different synthetic pathways for each type of HMOs.

It was noteworthy that there were complex relationships between HMOs and microorganisms. Previously studies reported that HMOs could directly mediate infant intestinal *Staphylococci, Streptococci, Lactobacilli and Enterococci,* or modulate maternal mammary epithelial cell responses and local immune responses (Bode 2012; Porro et al. 2022). Bifidobacterial proliferation in breastfed infants was correlated with the high levels of HMOs in breast milk from mothers (Le Doare et al. 2018; Moya-Gonzálvez et al. 2021). The breast milk was not considered as non-sterile and consisted of complex flora, which varies greatly among individuals (Tao et al. 2020). Based on the results from the measurements of colostrum samples, *Lactobacillus* and *Streptococcus* contents were higher compared with *Bifidobacterium* and *Staphylococcus*. The bacteria of breast milk could originate from the skin surface, and was associated with its own intestinal flora (Latuga et al. 2014). It was supposed that various bacteria utilized HMOs (such as *Bifidobacterium* and *Lactobacillus*), or benefited from their promotive/inhibitory effects without direct utilization (Craft and Townsend 2018; Zúñiga et al. 2018), with the possible relationships between HMOs and bacteria desired further exploration.

However, rare studies focused on the potential relationships between HMOs and bacteria of breast milk. The results from our study founded that DS-LNT was negatively related with *Staphylococcus*, which was consistent with the report that HMOs contained the bacteriostatic effect on pathogens from Yue et al. (2020). Rubio et al. (2019) also founded that the higher the level of *staphylococcus* in breast milk accompanied with the lower concentrations of total HMOs. Moossavi et al. (2019) analyzed the milk samples from 393 mothers and reported that oligosaccharides were associated with the diversity of lactic microbial communities with results including the negative relationship between HMOs and *Staphylococcus* (*r* = – 0.60 *p* = 0.038). It was believed that Staphylococci could bind to HMOs or HMOs contained the inhibitor effect on Staphylococci proliferation, which lead to the negative relationship between them. However, it was reported that the relative abundance of *Staphylococcus* was positively correlated with HMOs content based on the measurements from healthy breast milk samples (Williams et al. 2017). Therefore, the potential relationship still desired further confirmation through the clinical studies from with the expand sample size.

It was delighted that we found LNT was positively correlated with *Lactobacillus*. Rubio et al. (2019) also obtained the similar conclusion that higher levels of *Lactobacillus* were positively associated with concentrations of 2'-FL (*r* = 0.542, *p* = 0.038 in colostrum samples, and *r* = 0.700, *p* = 0.001 in mature samples). The results from our study proved that LNFP II, LNFP III, 3-SL, and LNnT possessed positive effects on *Streptococcus* proliferation. It was reported that *Streptococcus* levels were positively correlated with total HMOs in colostrum and transition samples of breast milk (Cabrera-Rubio et al. 2019). Besides, previously studies reported that oligosaccharides, such as 2'-FL et al., inhibited the growth of Group B Streptococci and *Streptococcus* pyogenes in vitro (Ackerman et al. 2017; Salli et al. 2020). However, the categories of *Streptococcus* genus also included the common probiotics such as *Streptococcus* thermophilus et al., in addition to Group B *Streptococcus* and *Streptococcus* pyogenes. It was important to conduct a series of studies to confirm the relationships between HMOs and *Streptococcus*. There were no relationships between HMOs and *Bifidobacterium* founded in our study. But evidences proved that *Bifidobacterium* was positively correlated with Sialylated HMOs and LNT (Aakko et al. 2017), and negatively correlated with DS-LNT (Moossavi et al. 2019). There were no associations between HMOs and bacteria of breast milk, which would be attributed to characteristic differences of HMO consumption by bacteria (Zúñiga et al. 2018). Therefore, the in-depth and comprehensive researches needed to be conducted to explore the potential relationships between HMOs and bacteria of breast milk.

In conclusion, there is a positive correlation between HMOs that depend on FUT2 in colostrum. We found that maternal clinical characteristics such as mode of delivery, number of delivery and lactation mode were associated with HMOs. In addition, there were correlations between HMOs and *Lactobacillus*, *Staphylococcus* and *Streptococcus* in milk. However, we were limited by multiple factors, such as the number of subjects, age, regional provinces and so on. In this study, secretor status was not determined on the basis of serological tests. In order to enrich the study of Chinese colostrum, it should be further studied in more groups in the future. Further experiments could be carried out to explore the mechanism of HMOs and Microbiota in vivo and in vitro.

## Data Availability

The data that support the findings of this study are available from the corresponding author upon reasonable request.
